# Even violins can cry: specifically vocal emotional behaviours also drive the perception of emotions in non-vocal music

**DOI:** 10.1098/rstb.2020.0396

**Published:** 2021-12-20

**Authors:** D. Bedoya, P. Arias, L. Rachman, M. Liuni, C. Canonne, L. Goupil, J.-J. Aucouturier

**Affiliations:** ^1^ Science and Technology of Music and Sound, IRCAM/CNRS/Sorbonne Université, Paris, France; ^2^ Department of Cognitive Science, Lund University, Lund, Sweden; ^3^ Faculty of Medical Sciences, University of Groningen, Groningen, The Netherlands; ^4^ Alta Voce SAS, Houilles, France; ^5^ BabyDevLab, University of East London, London, UK; ^6^ FEMTO-ST Institute, Université de Bourgogne Franche-Comté/CNRS, Besançon, France

**Keywords:** voice, music, emotions

## Abstract

A wealth of theoretical and empirical arguments have suggested that music triggers emotional responses by resembling the inflections of expressive vocalizations, but have done so using low-level acoustic parameters (pitch, loudness, speed) that, in fact, may not be processed by the listener in reference to human voice. Here, we take the opportunity of the recent availability of computational models that allow the simulation of three specifically vocal emotional behaviours: smiling, vocal tremor and vocal roughness. When applied to musical material, we find that these three acoustic manipulations trigger emotional perceptions that are remarkably similar to those observed on speech and scream sounds, and identical across musician and non-musician listeners. Strikingly, this not only applied to singing voice with and without musical background, but also to purely instrumental material.

This article is part of the theme issue ‘Voice modulation: from origin and mechanism to social impact (Part I)’.

## 1. Introduction

Originally invoked to describe the vocal monodic style of the Florentine Camerata in the seventeenth century [1], the idea that music expresses emotions by resembling the inflections of expressive speech (the so-called ‘speech theory’) has grown into a prominent view in recent psychological [2], neuroscientific [3] and evolutionary [4] accounts of music cognition. This view is notably supported by a wealth of studies showing that music’s expressive acoustic features mirror those used in vocal expression, with e.g. fast pace and high intensity for happy music/voice, and monotonous pitches and dark timbres for sad music/voice [5–8]. In addition, music and voice processing appear to obey similar innate developmental constraints, as shown, for example by comparable impairments in congenital amusia [9] or by improvements of prosodic perception after musical training [10].

It is unclear, however, whether these similarities reveal a genuine cross-domain recycling of cognitive resources developed originally either for voice or for music; or whether they reflect a mechanism that is simply more generic than either, and encompasses both. Voice and music cognition are indeed continuous with generic auditory cognition [11], and the majority of acoustic characteristics tested by prior work (e.g. pitch, loudness, speed) carry biologically significant information about a vaster diversity of sound sources than voice or music. For instance, abstract sound sources with increasing loudness and rising pitch may be perceived as gaining energy and moving closer, triggering avoidance reactions and a sense of urgency [12,13]. Similarly, adults, and infants as early as six months old, associate lower pitch with larger and potentially more formidable objects [14]. Accordingly, research has shown that changes in frequency, rate and intensity that are known to support emotional interpretations in speech and music in fact also trigger similar emotional responses when applied to environmental sounds such as rain, thunder or wind [15]. In addition, cross-domain contrasts in brain imaging of speech and music emotion typically do not reveal common sensory representations in temporal voice areas, as would be expected if these were voice-specific effects, but only supramodal emotion representations in the frontal cortices [16,17].

All of this suggests that the perceptual mechanisms so far tested in speech and music studies may not, in fact, be processed by the listener in reference to human voice. It remains unknown whether specifically vocal expressive cues, such as the unstable phonatory muscle control of an anxious voice, the nonlinear vocal fold vibration of a scream, or the bright resonating quality of smiled speech, also trigger comparable emotional reactions when they occur in music.

One reason previous research has not tested voice-specific cross-domain effects is the lack of tools able to simulate such phenomena in arbitrary audio material. First, typical acoustic manipulations in experimental stimuli have used generic audio processing software such as Audacity (Audacity Team) or ProTools (Avid Technology) [6,15], which only allow the transformation of low-level parameters such as pitch, intensity and speed. Second, voice-specific tools such as Praat [18] or SoundGen [19], which are able to model phonatory or articulatory aspects of human voice, do not allow transformation of musical excerpts in a way that mirror these characteristics.

**Figure 1.**
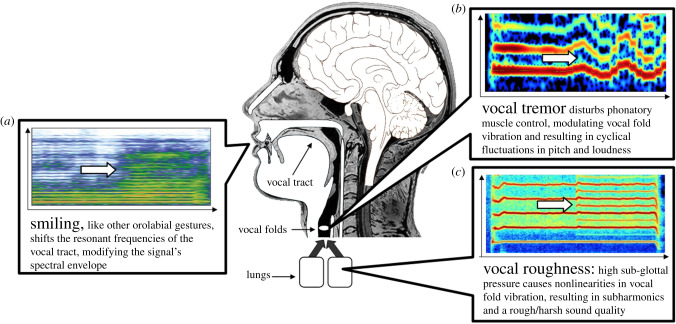
Three expressive acoustic changes that have a specifically vocal origin in the physiology of human/mammalian vocal apparatus: (*a*) smiling, (*b*) vocal tremor and (*c*) vocal roughness. All three changes are simulated here by signal processing techniques, which can modulate both speech and music recordings. (Online version in colour.)

Here, we take the opportunity of a series of recent developments in audio transformation technologies [20] that provide novel technical ways to simulate the effect of three voice-specific emotional behaviours (one articulatory, smiled speech [21]; two phonatory, vocal tremor [22] and vocal roughness [23]) identically in matched speech and music stimuli:

(i) Smiling, like other orolabial gestures such as nose wrinkling [24], modify the shape and length of the vocal tract [25], shifting its resonating frequencies (figure 1*a*). These changes can be simulated using frequency warping on the spectral envelope of the sounds, inside a phase vocoder architecture [21]. In listening experiments, English speech samples manipulated with such a transformation were validated to sound more smiling, and generally more positive [21,26]; in production experiments, participants asked to imitate voices manipulated with such changes do so by smiling while they vocalize [26].
(ii) Vocal tremor, which can occur physiologically from cold, fatigue or anxiety, is a rhythmical and involuntary oscillatory movement affecting the vocal folds, thought to result from disturbances in the neurophysiological feedback processes of phonatory muscle control [27,28]. It causes cyclical fluctuations in pitch (vibrato, figure 1*b*) and loudness (tremolo), which can be simulated in recordings as the sinusoidal modulation of a pitch shift effect [22]. In listening experiments, English, French, Swedish and Japanese speech samples manipulated with such a transformation were validated to sound more anxious, negative and aroused [22,29]; in production experiments, participants who heard themselves speak while their auditory feedback was manipulated with tremor reported feeling more negative and more aroused [29].
(iii) Vocal roughness, which occurs when excessive subglottal pressure due to effort or arousal causes nonlinearities in vocal fold vibration, reveals the presence in voice of subharmonics (figure 1*c*), which, along with other nonlinearities such as frequency jumps, broadband noise or chaos, gives voice a rough and noisy quality [30]. Vocal roughness in screams, cries, grunts or moans has an important communicative function in the human expressive repertoire, because it signals aversive states such as fear, pain or distress [31,32]. Vocal roughness can be simulated using pitch-synchronous amplitude modulation to add subharmonics in the original signal [23]. In listening experiments, speech samples manipulated with such a transformation were validated to sound more negative and aroused [23].

Using such manipulations designed in clear mechanistic analogy with the human voice is important because it ensures that we only explore a range of acoustic variations that correspond to what voice can do (e.g. smiling operates on the 2–4 kHz frequency range, and not, say, at 1 or 8 kHz), at a level of intensity that conforms to daily ‘mundane’ expressions (e.g. a pitch shift of +25 cents, a quarter of a semitone, and not, say, +3–4 semitones), and avoid broad claims of similarity based on sound manipulations (e.g. a wholesale +5 semitones applied to a complete orchestral piece) that, in fact, may not be processed by the listener in reference to human voice.

In this work, we applied all three vocal manipulations to matched speech, vocal music and instrumental music extracts. We asked two groups of *N* = 29 musician and *N* = 31 non-musician listeners to compare pairs composed of the manipulated and non-manipulated variants of each sound using two Likert scales for expressed emotional valence and arousal, and examined whether the manipulations led to similar emotional interpretations when they occurred in speech and music. Ratings of valence and arousal were chosen in order to measure the low-level expression of ‘core affect’ [33], which is more likely to capture affective similarities between speech and music pairs than higher-level categorical constructs such as emotions, which are expected to be more heavily influenced by context such as the presence or the absence of lyrics [34] or of a specific musical instrument [35].

## 2. Results

### (a) Preregistered hypotheses

We tested the impact of the three manipulations (smiling, vocal tremor and vocal roughness) on five types of sounds: two types of non-musical vocal sounds (speech and screams), and three types of musical sounds (singing only, singing + music, violin + music).

In the following, we separately report, for each of the three manipulations, on five-level analyses including all these types of sounds. However, our hypotheses, which we preregistered (https://aspredicted.org/mc72i.pdf), concerned only a subset of these combinations:

(i) Smiling and vocal tremor are manipulations originally developed and validated for speech sounds [21,22]. Following these studies, we hypothesized that smiling would increase valence and arousal, and vocal tremor would decrease valence and increase arousal for speech stimuli. We made no hypotheses for how these manipulations would affect the perception of screams.
(ii) Conversely, vocal roughness is a manipulation originally developed and validated for screams [23]. Following this study, we hypothesized that roughness would decrease valence and increase arousal for scream sounds. We made no hypothesis for how vocal roughness would affect the perception of speech.
(iii) Similarly, our hypotheses concerning the transfer of affective qualities from non-musical vocal sounds (speech and screams) to musical sounds concerned speech effects for smiling and vocal tremor (i.e. similar to speech, smiling would increase valence and arousal for musical sounds, and vocal tremor would decrease valence and increase arousal) and scream effects for vocal roughness (i.e. similar to screams, vocal roughness would decrease valence and increase arousal for musical sounds).

### (b) The three manipulations worked as intended on vocal sounds

We first validated that the three voice manipulations triggered emotional judgements as intended when occurring on vocal sounds. *N* = 60 participants (among whom *N* = 29 were musicians) rated pairs of matched manipulated and non-manipulated sounds on both valence and arousal. As preregistered, we aggregated participant ratings for each type of stimulus and transformation, and analysed the effect of transformation using repeated-measure ANOVAs and paired *t*-tests.

(i) The effect of applying the smile transformation (smile versus unsmile) to speech stimuli was very large and statistically significant: as predicted, it led to higher perceived valence (*M* = +1.01, [+ 0.79, +1.24] scale points, *t*_59_ = 9.09, *p* = 8.00 × 10^−13^, Cohen’s *d* = 1.92) and perceived arousal (*M* = +1.27, [1.02, 1.53], *t*_59_ = 10.08, *p* = 1.89 × 10^14^, *d* = 2.09). Neither of these effects interacted statistically with participants being musicians or not (interaction musician × transformation, valence: *F*_2,116_ = 1.23, *p* = 0.30, $\eta_{p}^{2}=0.02$; arousal: *F*_2,116_ = 2.40, *p* = 0.10, $\eta_{p}^{2}=0.04$; test sensitive to effect size *d* ≥ 0.28 at power 1 − *β* = 0.95 and *α* = 0.05).
(ii) The effect of applying the tremor transformation (tremor versus non-manipulated) to speech stimuli was medium and statistically significant. As expected, it decreased perceived valence (*M* = −0.19, [−0.28, −0.11], *t*_59_ = −4.55, *p* = 2.77 × 10^5^, *d* = 0.59). However, contrary to what we predicted, tremor also decreased perceived arousal (*M* = −0.19, [− 0.27, − 0.11], *t*_59_ = −4.88, *p* = 8.56 × 10^−6^, *d* = 0.55). Neither of these effects interacted statistically with participants being musicians or not (interaction musician × transformation, valence: *F*_1,58_ = 2.62, *p* = 0.11, $\eta_{p}^{2}=0.04$; arousal: *F*_1,58_ = 0.03, *p* = 0.87, $\eta_{p}^{2}=0.00$; test sensitive to effect size *d* ≥ 0.31 at power 1 − *β* = 0.95 and *α* = 0.05).
(iii) The effect of applying the roughness transformation (rough versus non-manipulated) to scream stimuli was very large and statistically significant. As expected, it decreased perceived valence (*M* = −0.71, [−0.89, −0.53], *t*_59_ = −7.78, *p* = 1.28 × 10^−10^, *d* = 1.30) and increased arousal (*M* = +0.62, [0.45, 0.8], *t*_59_ = 7.09, *p* = 1.90 × 10^−9^, *d* = 1.21). Neither of these effects interacted statistically with participants being musicians or not (valence: *F*_1,58_ = 0.94, *p* = 0.34, $\eta_{p}^{2}=0.02$; arousal: *F*_1,58_ = 0.27, *p* = 0.60, $\eta_{p}^{2}=0.00$; test sensitive to effect size *d* ≥ 0.31 at power 1 − *β* = 0.95 and *α* = 0.05).

In sum, the effects of the three manipulations were largely consistent with our predictions for vocal sounds. Descriptively, the effect of smiling on speech was consistent with expressing more positivity and arousal, tremor on speech with expressing more negativity and less arousal (note that previous work associated tremor with increased, rather than decreased, arousal [22,29]) and roughness on screams with expressing more negativity and more arousal.

### (c) Extension to non-preregistered vocal modes

**Figure 2.**
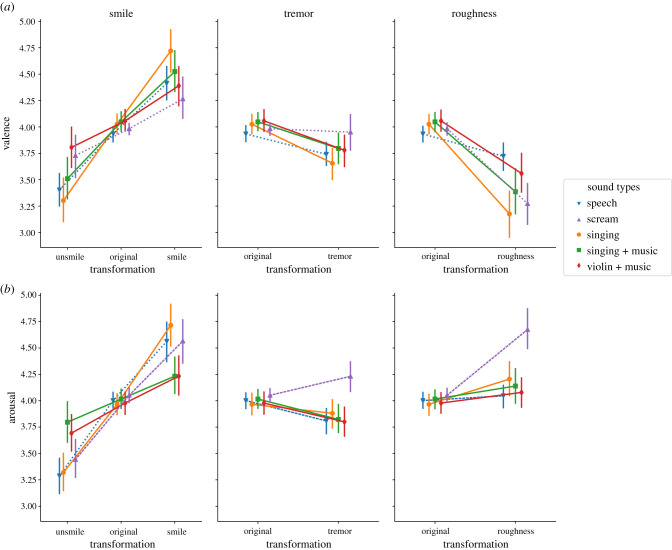
Vocal manipulations of smiling, tremor and roughness trigger similar emotional perceptions on both vocal and non-vocal music. Valence (*a*) and arousal (*b*) ratings for smiling, vocal tremor and vocal roughness manipulations of matched vocal (speech, scream; dashed lines) and musical stimuli (solid lines). For each manipulation and each sound type, ratings are given both for manipulated pairs (12–14 pairs consisting of one manipulated sound, evaluated in comparison with its non-manipulated variant; labelled as ‘smile’, ‘tremor’, etc.) and for control pairs (12–14 pairs consisting of one non-manipulated sound, evaluated in comparison with itself; labelled as ‘original’). Error bars indicate 95% confidence intervals on the mean. (Online version in colour.)

Even though we only preregistered hypotheses for smile and tremor on speech, and for roughness on screams (respecting the vocal modes for which the manipulations were originally intended), all three manipulations were also tested for the other vocal mode:

(i) The effect of smiling on screams was consistent with predictions made for speech (valence: *M* = +0.53, [0.26, 0.81], *t*_59_ = 3.88, *p* = 0.0003, *d* = 0.77; arousal: *M* = +1.13 [0.86, 1.39], *t*_59_ = 8.37, *p* = 1.32 × 10^−11^, *d* = 1.68).
(ii) Contrary to speech, tremor had no effect on the valence of screams (*M* = −0.04, [−0.19, 0.12], *t*_59_ = −0.45, *p* = 0.65, *d* = 0.07) and increased their perceived arousal (*M* = +0.18, [0.07, 0.3], *t*_59_ = 3.14, *p* = 0.002, *d* = 0.46; note, prospectively, that the effect of tremor on scream arousal was in an opposite direction to all other sound types) (figure 2).
(iii) Finally, the effect of roughness on speech was consistent with predictions made for screams, decreasing valence (*M* = −0.21, [−0.33, −0.09], *t*_59_ = −3.45, *p* = 0.001, *d* = 0.54) and increasing arousal, albeit non-significantly (*M* = +0.05, [−0.04, 0.13], *t*_59_ = 1.12, *p* = 0.26, *d* = 0.14).

### (d) All voice manipulations had a similar effect on vocal and instrumental musical sounds

The same *N* = 60 participants then rated manipulated pairs of matched musical sounds in three conditions: singing only (‘*a cappella*’ recording reproducing the same verbal content as the speech stimuli), singing + music (manipulated singing track, mixed with non-manipulated instrumental background) and violin + music (manipulated violin track recorded to imitate the singing track, mixed with non-manipulated instrumental background).

To avoid demand effects, participants rated the music pairs before rating the speech and scream pairs used for validation above; all three types of musical sounds and three types of transformations were randomized within the music block; participants were unaware of the possibility of algorithmic manipulation; and pairs of identical stimuli were included for control (similar procedure as [15], see Material and methods).

All three vocal manipulations triggered emotional judgements on musical stimuli that were strikingly similar to those observed on vocal stimuli (figure 2):

(i) The 5-level sound-type factor interacted significantly with the effect of smile on valence (*F*_8,472_ = 11.58, *p* = 4.60 × 10^−15^, $\eta_{p}^{2}=0.16$) and arousal (*F*_8,472_ = 15.57, *p* = 2.12 × 10^−20^, $\eta_{p}^{2}=0.21$), but all effects were in the same direction. Our prediction for transfer to musical sounds concerned the effect of smiling on speech: similarly to speech, the smile manipulation increased the perceived valence and arousal when applied to *a cappella* singing (valence: *M* = +1.45, [1.14, 1.75], *t*_59_ = 9.56, *p* = 1.37 × 10^−13^, *d* = 2.07; arousal: *M* = +1.41, [1.14, 1.67], *t*_59_ = 10.50, *p* = 4.05 × 10^−15^, *d* = 2.17), and to singing mixed with instrumental background (valence: *M* = +1.02, [0.76, 1.28], *t*_59_ = 7.89, *p* = 8.56 × 10^−11^, *d* = 1.55; arousal: *M* = +0.44, [0.23, 0.65], *t*_59_ = 4.16, *p* = 1.06 × 10^−4^, *d* = 0.76), but also when applied to a non-vocal (violin) track mixed with instrumental background (valence: *M* = +0.57, [0.35, 0.8], *t*_59_ = 5.13, *p* = 3.43 × 10^−6^, *d* = 0.89; arousal: *M* = 0.54, [0.33, 0.74], *t*_59_ = 5.30, *p* = 1.82 × 10^−6^, *d* = 0.93). In short, as for speech, violin made to sound more smiling was perceived as more positive and more aroused.
(ii) The 5-level sound-type factor interacted significantly with the effect of tremor on valence (*F*_4,236_ = 3.72, *p* = 5.90 × 10^−3^, $\eta_{p}^{2}=0.06$) and arousal (*F*_4,236_ = 9.37, *p* = 4.78 × 10^−7^, $\eta_{p}^{2}=0.14$) but, again, all effects were in the same direction (except for the non-preregistered case of scream arousal). Our prediction for transfer to musical sounds concerned the effect of tremor on speech: similarly to speech, the tremor manipulation decreased the perceived valence and arousal (the latter non-significantly) when applied to *a cappella* singing (valence: *M* = −0.37, [−0.51, −0.22], *t*_59_ = −5.09, *p* = 3.89 × 10^−6^, *d* = 0.85; arousal: *M* = −0.08, [−0.22, 0.05], *t*_59_ = −1.20, *p* = 2.37 × 10^−1^, *d* = 0.19), decreased both significantly when applied to singing + music (valence: *M* = −0.26, [−0.39, −0.12], *t*_59_ = −3.86, *p* = 2.87 × 10^−4^, *d* = 0.59; arousal: *M* = −0.19, [−0.3, −0.09], *t*_59_ = −3.80, *p* = 3.41 × 10^−4^, *d* = 0.50) and to violin + music (valence: *M* = −0.28, [−0.41, −0.14], *t*_59_ = −3.99, *p* = 1.84 × 10^−4^, *d* = 0.62; arousal: *M* = −0.19, [−0.31, −0.06], *t*_59_ = −3.04, *p* = 3.48 × 10^−3^, *d* = 0.42). In short, as for speech, violin made to sound more trembling was perceived as less positive and less aroused.
(iii) The 5-level sound-type factor interacted significantly with the effect of roughness on valence (*F*_4,236_ = 12.70, *p* = 2.25 × 10^−9^, $\eta_{p}^{2}=0.18$) and arousal (*F*_4,236_ = 13.57, *p* = 5.69 × 10^−10^, $\eta_{p}^{2}=0.19$) but, again, all effects were in the same direction. Our prediction for transfer to musical sounds concerned the effect of roughness on screams: similarly to screams, the roughness manipulation decreased valence and increased arousal when applied to *a cappella* singing (valence: *M* = −0.85, [−1.08, − 0.61], *t*_59_ = −7.24, *p* = 1.05 × 10^−9^, *d* = 1.33; arousal: *M* = +0.24, [0.07, 0.41], *t*_59_ = 2.77, *p* = 7.49 × 10^−3^, *d* = 0.49), and decreased valence when applied to singing + music (valence: *M* = −0.66, [−0.87, −0.45], *t*_59_ = −6.17, *p* = 6.83 × 10^−8^, *d* = 1.05) and to violin + music (valence: *M* = −0.49, [−0.68, −0.31], *t*_59_ = −5.27, *p* = 2.02 × 10^−6^, *d* = 0.87). The effect of vocal roughness on arousal on singing + music and violin + music was also in the expected direction, but non-significantly (singing+music: *M* = +0.13, [−0.02, 0.27], *t*_59_ = 1.74, *p* = 0.09, *d* = 0.28; violin + music: *M* = +0.10, [−0.03, 0.23], *t*_59_ = 1.51, *p* = 0.13, *d* = 0.22). In short, as for screams, violin made to sound rougher was perceived as less positive and more aroused.

### (e) Effects were larger on isolated singing than with musical accompaniments

**Figure 3.**
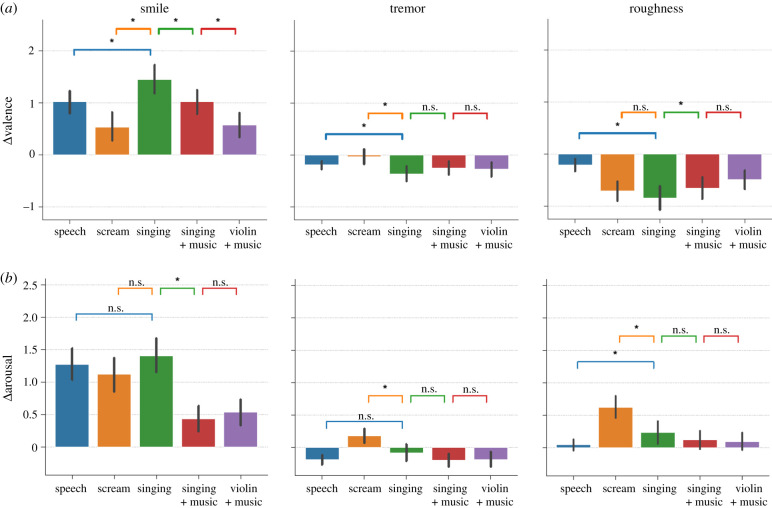
The effect of vocal manipulations on isolated singing (green) was similar to or larger than on spoken voice (blue, orange), but was smaller on instrumental music (red, purple). Normalized ratings (smile: smile − unsmile; tremor: tremor − original; roughness: rough − original) for valence (*a*) and arousal (*b*) of the smiling, vocal tremor and vocal roughness manipulations in each type of stimulus. Asterisks indicate statistical significance of pairwise *t*-tests at the *p* < 0.05 level. Error bars indicate 95% confidence intervals on the mean. (Online version in colour.)

Even though all emotional perceptions in manipulated musical sounds were in the same direction as for vocal sounds, there were differences in the intensity of these perceptions, as indicated by statistical interactions between manipulation and sound type (figure 3):

(i) The 5-level sound type interacted with the effect of smiling on both perceived valence (*F*_4,236_ = 14.93, *p* = 6.83 × 10^−11^, $\eta_{p}^{2}=0.20$) and arousal (*F*_4,236_ = 21.11, *p* = 6.81 × 10^−15^, $\eta_{p}^{2}=0.26$).
  For valence, the effect of smiling was larger on speech (*d* = 1.92) than on screams (*d* = 0.77, *t*_59_ = −3.35, *p* = 0.001). Within musical sounds, it was maximal on singing voice (*d* = 2.07), on which it was larger than on speech (*t*_59_ = 3.23, *p* = 0.002) and screams (*t*_59_ = 5.44, *p* < 0.00001). Compared with singing, the effect of smiling was smaller on singing + music (*d* = 1.55; *t*_59_ = −4.17, *p* < 0.00001) and smaller again (but remained large) on violin + music (*d* = 0.89; *t*_59_ = −6.33, *p* < 0.00001).
  For arousal, the effect of smiling did not differ between speech (*d* = 2.09), screams (*d* = 1.68; *t*_59_ = 1.21, *p* = 0.23) and singing (*d* = 2.17; *t*_59_ = 0.89, *p* = 0.37). It was smaller (but remained large) on singing + music (*d* = 0.76; *t*_59_ = −8.87, *p* < 0.00001) and on violin + music (*d* = 0.93; *t*_59_ = −6.60, *p* < 0.00001; figure 3, left) than on singing.
(ii) The 5-level sound type interacted with the effect of tremor on both perceived valence (*F*_4,236_ = 3.72, *p* = 0.0059, $\eta_{p}^{2}=0.06$) and arousal (*F*_4,236_ = 9.37, *p* = 4.78 × 10^−7^, $\eta_{p}^{2}$), but these interactions were merely driven by the difference between speech and screams (for which tremor had no effect on valence and an opposed effect on arousal).
  For valence, the effect of tremor was marginally larger (more negative) on speech (*d* = 0.59) than on screams (*d* = 0.07; *t*_59_ = 1.76, *p* = 0.083). Within musical sounds, the valence effect of tremor was maximal (i.e. more negative) on singing (*d* = 0.85), on which it was larger than speech (*t*_59_ = 2.19, *p* = 0.033) and screams (*t*_59_ = 2.95, *p* = 0.005). Compared with singing, the valence effect of tremor was not significantly smaller on singing + music (*d* = 0.59; *t*_59_ = −1.49, *p* = 0.14) or on violin + music (*d* = 0.62; *t*_59_ = −0.94, *p* = 0.35).
  For arousal, the effect of tremor was significantly different, and in opposed directions, on speech (less arousal, *d* = 0.55) and screams (more arousal, *d* = 0.46, *t*_59_ = 5.64, *p* < 0.00001). Within musical sounds, none of the arousal effects was of significantly different amplitude than on speech (singing: *d* = 0.19, *t*_59_ = −1.76, *p* = 0.08; singing + music: *d* = 0.50, *t*_59_ = −0.05, *p* = 0.96; violin + music: *d* = 0.42, *t*_59_ = −0.09, *p* = 0.93), nor did they differ from one another (all *p*-values >0.21). All differed significantly from screams (singing: *t*_59_ = 3.12, *p* = 0.003; singing + music: *t*_59_ = 5.27, *p* < 0.00001; violin + music: *t*_59_ = 5.17, *p* < 0.00001; figure 3, middle).
(iii) The 5-level sound type interacted with the effect of roughness on both perceived valence (*F*_4,236_ = 12.70, *p* = 2.25 × 10^−9^, $\eta_{p}^{2}=0.18$) and arousal (*F*_4,236_ = 13.57, *p* = 5.69 × 10^−10^, $\eta_{p}^{2}=0.19$).
  For valence, the effect of vocal roughness was maximum on singing voice (*d* = 1.33) and screams (*d* = 1.30; no statistical difference: *t*_59_ = 1.20, *p* = 0.23). It was smaller than on singing (but remained large) on singing + music (*d* = 1.05; *t*_59_ = −2.85, *p* = 0.006) and on violin + music (*d* = 0.87; *t*_59_ = −3.50, *p* = 0.001).
  For arousal, the effect of vocal roughness was maximum on screams (*d* = 1.21), for which it was larger than on speech (*d* = 0.14; *t*_59_ = 6.36, *p* < 0.00001). Within musical sounds, the effect of roughness was smaller than on screams, singing (*d* = 0.49; *t*_59_ = −3.47, *p* = 0.001), singing + music (*d* = 0.28; *t*_59_ = −5.17, *p* < 0.00001) and violin + music (*d* = 0.22; *t*_59_ = −4.84, *p* < 0.00001; figure 3, right).

### (f) No effect of musicianship

**Figure 4.**
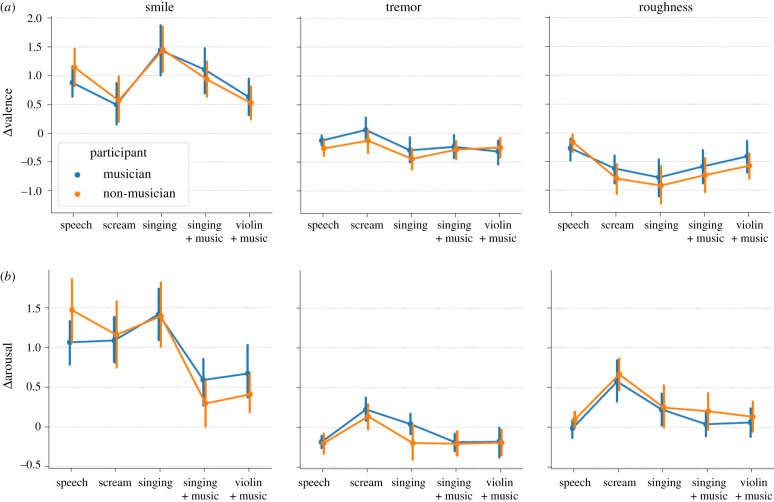
No interaction of musicianship on the effect of sound type on normalized valence and arousal, for any of the manipulations. Normalized ratings (smile: smile − unsmile; tremor: tremor − original, roughness: rough − original) for valence (*a*) and arousal (*b*), in the musician (blue) and non-musician (orange) groups. Error bars indicate 95% confidence intervals on the mean. (Online version in colour.)

Finally, to examine whether participant musicianship interacted with the effects, we computed normalized valence and arousal ratings (smile: smile − unsmile; tremor: tremor − original, roughness: rough − original) and averaged over all stimuli per participant and sound type. Whether participants were self-declared musicians (*N* = 29) or non-musicians (*N* = 31) did not interact with the effect of sound type on normalized valence and arousal, for any of the manipulations (all *p*-values >0.49, except smiling arousal: *F*_4,232_ = 2.24, *p* = 0.066, $\eta_{p}^{2}=0.04$; figure 4; test sensitive to effect sizes *d* ≥ 0.23 at power 1 − *β* = 0.95 and *α* = 0.05).

## 3. Discussion

A wealth of theoretical and empirical arguments have suggested that music triggers emotional reactions by resembling the inflections of expressive vocalizations, but past research focused on low-level acoustic parameters (pitch, loudness, speed) which, in fact, may not be processed by the listener in reference to human voice. Here, we provided a more direct test of the hypothesis by using computational voice-transformation models that simulate three emotional behaviours linked to specifically vocal mechanisms of articulation (smiling) and phonation (vocal tremor and vocal roughness). When applied to musical material, we found that these three highly specific acoustic manipulations triggered emotional perceptions that were remarkably similar to those observed for speech and scream sounds. Strikingly, this applied not only to singing voice with and without musical background, but also to purely instrumental material: even violins can cry, or at least sound more positive and aroused when smiling, more negative and less aroused when trembling, and more negative when screaming (figure 2).

Importantly, while they can be simulated using inanimate, non-vocal artefacts (e.g. a dented clay cylinder for smile [25]; a periodically rotating sound source for vocal tremor [36]), none of the three behaviours tested here has non-vocal ecological equivalents in nature, because they closely depend on the dynamics and physiology of the mammalian larynx: smiling is a dynamic change of resonating frequencies of the vocal tract, vocal tremor is an extrinsic modulation of the vocal folds of muscular-control origins, and vocal roughness is the consequence of a nonlinear regime of vocal fold oscillation. If these changes also impart emotional qualities when they occur in music, then these must therefore necessarily be of human (or animal) vocal origin. Our results thus provide the literal confirmation of Darwin’s conjecture that musical emotions can stem from acoustic features that resemble ‘the voices of other animals and man’s own instinctive cries’ [37].

Even though all emotional perceptions in manipulated musical sounds were in the same direction as vocal sounds, there were differences in the intensity of these perceptions, among both musical and non-musical sounds. Among non-musical sounds (speech and screams), smiling and tremor both had greater effects (respectively positive and negative) on perceived valence in speech than in screams; conversely, vocal roughness had a more negative effect on the perceived valence of screams than of speech, and no arousing effect on speech. These differences between speech and screams are likely explained by discrepancies between the emotional valence of the changes and the vocal context in which they occur. For instance, while smiling can signal dominance [38], it is not typically associated with screamed vocalizations and therefore plausibly warrants less univocally positive interpretations in this context than on spoken voice. Similarly, while vocal tremor in vocal registers with low subglottal pressure is typically associated with negative evaluations of e.g. sadness or stress [22,27], the same pitch oscillations when heard in screamed stimuli may be associated with nonlinearities due to high subglottal pressure (e.g. pitch jumps) and attributed to higher arousal or intensity rather than lower valence [39]; and, in a similar manner, vocal roughness, while indicative of arousal and aversiveness in screams, may be attributed in the low-pressure register of spoken voice to non-emotional phenomena such as vocal fatigue or hoarseness [40,41]. Finally, it should be noted that the effect of vocal tremor on arousal was in a different direction for speech (negative) and for screams (positive; figure 2, middle-bottom). That speech effect was the only effect found in a direction that we did not predict. Because the effect was negative for both speech and music, it is plausible that the low-arousal effect of tremor is a genuine effect that transferred from speech to music (our main hypothesis), but it also remains possible that the tremor effect on speech is due to a learning effect carried over from the (previously judged) musical pairs, which would have been evaluated differently had the speech pairs been presented in isolation.

Among musical sounds, the effect of the three manipulations was generally larger for *a cappella* singing voice than for non-musical vocalizations (speech or scream): this was true for the effect of smile, vocal tremor and, to some extent, vocal roughness on valence (but not on arousal). It is possible that the acoustical properties of singing voice [42] benefit the perception of the three cues used here. For instance, musical melody in the contemporary commercial music genres considered here features discrete and relatively stable pitch series which, as opposed to the continuously changing pitch of speech intonation [43], may facilitate the processing of slowly-changing pitch modulations in vocal tremor. Further, the fact that sung vowels and consonants are typically longer than in their normal occurrence in speech [44] may also allow the faster accumulation of spectral/harmonic information to register changes like smile or vocal roughness. Such an explanation may be conceptually related to the ‘super-expressive voice hypothesis’, a prominent theory of musical emotions stating that, because of their wider pitch and dynamic range, music may be processed as amplified and exaggerated vocal expressions, resulting in more intense emotional reactions [2,5]. It is possible that, even when manipulation intensity is controlled to be strictly identical as for speech, the specific acoustics of singing voice may provide a clearer, more contrasting background for emotional expression than connected speech.

On the other hand, while our three manipulations were qualitatively similar on vocal and instrumental music, they were not perceived as more intense on non-vocal musical instruments than on human voice (if anything, they were even less intense). Among musical sounds, the effect of the three manipulations was indeed greater for *a cappella* singing than for music with instrumental background. One possible explanation is perceptual, as the additional instrumental background may create masking effects that make registering the (relatively subtle) changes of the main track more difficult. For instance, smiling is a spectral manipulation mostly manifest in the high–medium frequency range of formants F2–F5 (600–3500 Hz) [45], which is a frequency band likely to be already crowded in the instrumental mixes of the popular music genres tested here. Similarly, the perception of vocal roughness involves the registering of irregularities in the harmonicity of the source (i.e. subharmonics), which may be hindered in the presence of a harmonic musical background [46]. Another possible explanation is psychological, where the emotional quality of the manipulated vocal source may be dampened because of its superposition with a non-manipulated and possibly non-emotionally-congruent background. In the present work, participants were instructed to rate the expression perceived in music as a whole, and not e.g. of a specific vocal source while ignoring the background [46], which may have also contributed to these effects. Finally, the explanation may also be technical, owing to the possibly limited applicability of the transformation algorithms to non-vocal material. The fact that we did not present participants with a solo-instrument condition (without concurrent musical background) is limiting our ability to arbitrate between these possibilities, and could be considered for future work.

While the fact that singing voices can be expressively smiling, trembling or screaming may not appear surprising from a naturalistic, biological point of view, and is in accordance with comparative acoustic analyses of emotion production in speech and singing [42], it strongly contrasts with an ‘artificialistic’ view, prevalent for instance in the musicology of the great virtuoso performers of the nineteenth century [47], of singing voice as a disembodied musical instrument bearing no natural relation to the singer’s body [48]. The present results suggest, on the contrary, that singing and non-vocal musical sounds can both be processed *as if* they were spoken voice, mobilizing cognitive mechanisms linked to the detection and interpretation of physiological phenomena. The violin stimuli used here were artificially constructed using voice-specific gestures and one may question their ecological validity, i.e. whether musicians can actually manipulate these aspects of their sounds. Many elements suggest they can. First, there are well-described acoustic similarities between the human voice and violin [49,50], which has a similar frequency range and a formant structure exhibiting vowel-like qualities [51], leading many to describe violin playing as sounding either male (‘He had a stroke so sweet, and made it speak like the voice of a man’ [52, p. 154]) or female (‘There are in the music of the violin—if one does not see the instrument itself […]—accents which are so closely akin to those of certain contralto voices, that one has the illusion that a singer has taken her place amid the orchestra’ [53, p. 378]). Second, many traditional violin gestures can be said to ressemble the source-filter parameters manipulated in this work: while violin strings are ordinarily bowed or plucked in the centre of the fingerboard, violinists intentionally bow strings at the other positions (e.g. close to the bridge: *sul ponticello*) to create variations in timbre, which may resemble the type of gesture found in smiling, or nasality [54]; vibrato is commonly produced by oscillating the left hand around the position where it stops the string against the fingerboard and, while typically slower, is a clear parent to singing vibrato and vocal tremor [55] (‘It’s particularly interesting that it’s singing that violin playing has always been said to imitate, with violinists considered the divas of instrumental playing. The ease with which a violinist produces portamento and vibrato is, of course, the main reason’ [50, ch. 5, para. 51]). Finally, in contemporary performance, high bow pressure can be used to create distortion and ‘scratching’ sounds that may resemble vocal roughness [56]. Similar gestures are also found in other instruments, such as controlling brightness in brass instruments by employing slight changes in embouchure, akin to smiling [57], or saturated electrified instruments, for which acoustic similarities to rough alarm calls have been studied in the field of animal communication [58]. All these examples suggest that cultural evolution has found ways, by virtue of innovations in organology, performance or repertoire, to map the natural expressive resources of spoken voice to musical parameters, and ritualize them into musical practice.

Furthering this idea, we tested two groups of (self-reported) musicians and non-musicians. A wealth of empirical evidence has shown that musical training enhances auditory and pitch processing [59] and the ability to recognize emotions in music [60], and that these effects transfer to recognizing emotions in speech [10,61,62]. It could therefore be expected that musicians should perform differently from non-musicians, either because of an enhanced ability to perceive subtle vocal cues in complex music mixes, because of greater familiarity with e.g. the instrumental timbre of the violin, or because of a different cultural understanding of cues like vibrato or spectrum. We found no evidence that it was the case: whether participants were self-declared musicians or non-musicians did not interact with the effect of the manipulations, in any of the sound types tested here. This pattern of results reinforces the notion that, when applied to musical material, the three acoustic manipulations considered here do not operate as domain-specific conventions, but are rather founded in natural vocal expression. Note, however, that it is questionable whether a small, 3-years-of-musical-practice difference between groups can elicit such behavioural variation, and future work should consider better-controlled measures of musical ability before issuing strong conclusions about individual differences in how vocal expressions are perceived in music.

Finally, the work reported here is purely behavioural, and involves explicit ratings. From this sole comparison of vocal and musical expression, it is difficult to judge the extent to which the two types of processing are similar: they could involve similar sensorimotor representations (in effect hearing smiling violins *as if* they were smiling), or different representations converging at the same evaluation. Further work could attempt to clarify the sensory and cognitive mechanisms involved in the evaluation of specifically vocal changes on non-vocal sources such as violins using adaptation paradigms with voice–instrument hybrid sources [63,64] or implicit sensorimotor paradigms such as facial mimicry (e.g. does one imitate a smiling violin? [26]). It is also an open question whether the same sound variations would impart the same emotional effects in non-vocal natural sounds [15]. Even if the acoustic signatures considered here can be found elsewhere and have non-vocal origins (e.g. roughness in the rumble of thunder, or fluctuations of brightness in the coloured noise of wind), it is still possible that our multimodal (audiovisual, proprioceptive, etc.) experience of similar signatures in voices gives meaning to these otherwise meaningless sound variations.

It also remains unknown whether the almost transparent transfer of vocal parameters to non-vocal musical sounds demonstrated here applies to all music, or all experiences of music. It is probable that vocal cues only drive expressivity for music that bears some amount of analogy to human vocalization, making it possible to hear it ‘as if’ it was voice [1]. This is notoriously the case for violin, as already noted, and it would therefore be interesting to test whether these results extend to other musical instruments. It is also possible that some of the present results depend on the specific music genres (contemporary commercial music) used in this study. This may be especially true of vocal tremor, which is found here to be congruent (more negative, less aroused) in both speech and music, while previous research with operatic singers has found discrepancies between the use of speech vibrato associated with sadness (like here) and sung vibrato with anger (unlike here, i.e. greater rather than lower arousal) [42]. More generally, the mechanism identified here is plausibly only one of a plurality of ways by which music can be expressive. Musical emotions are shaped by cultural-evolutionary processes occurring in a great diversity of contexts, which are likely to take biological foundation in not only communicative adaptations such as vocal signalling, but also expressive motion [65], environmental monitoring [15], coalitional interactions, infant care [66], and others. It is now important to understand how these mechanisms interact with each other to shape our emotional musical experiences.

## 4. Material and methods

### (a) Participants

Here *N* = 60 participants (*M* = 23.1 years old, s.d. = 3.2; female: 31) took part in the experiment. *N* = 29 identified as musicians (more than 3 years of formal musical practice) and *N* = 31 as non-musicians (no formal musical practice). All participants reported normal hearing, normal or corrected-to-normal vision and no neurological or psychiatric disorder.

### (b) Auditory stimuli

We selected 14 excerpts from songs of various popular music genres (pop, jazz, rock), available as unmixed, multi-track recordings from the free online resource ‘Mixing Secrets For The Small Studio’ (http://www.cambridge-mt.com/ms-mtk.htm). For each recording, we selected one full musical phrase (singing + accompaniment) of average duration *M* = 7 s.

For each excerpt, we then used the available multi-tracks to create variants in four conditions: singing (the lead vocal track, without instrumental accompaniment), singing + accompaniment (the original song, composed of lead vocal track and instrumental accompaniment), violin + accompaniment (the original song, in which the lead vocal track was replaced by a violin instrumental track matching the main melody) and speech (a recording of a transcription of the lyrics of the lead vocal track, performed as non-musical speech). None of the 14 accompaniment tracks in conditions ‘singing + accompaniment’ and ‘violin + accompaniment’ contained additional background vocals.

The instrumental track in the ‘violin + accompaniment’ condition was recorded on the violin by a semi-professional musician in overdubbing conditions matching the pitch and phrasing of the original vocal track. Speech tracks in the ‘speech’ condition were recorded by two native English speakers (one male, one female, matching the gender of the original singer), who performed a spoken, neutral-tone rendition of the lyrics, without knowing or hearing that these were originally singing material. All recordings were performed in music production studios in IRCAM (Paris, France) by a professional sound engineer (D.B.). In addition, we also selected 12 ‘scream’ stimuli from a previous study [23], which consisted of short, isolated shouts of phoneme /a/, recorded by six male and six female actors. These resulted in 68 sets of multi-track stimuli, matched in five different conditions (speech: 14; singing: 14; singing + accompaniment: 14; violin + accompaniment: 14; and an unmatched set of 12 screams).

Before mixing, the lead track (vocal in conditions ‘speech’, ‘screams’, ‘singing’, ‘singing + accompaniment’; violin in condition ‘violin + accompaniment’) in each of the multi-track stimuli was then processed with three acoustic manipulations simulating specifically-vocal behaviours: smiling (two levels: *smile* and *unsmile*), vocal tremor (one level: *tremor*) and vocal roughness (one level: rough). Finally, the tracks of each stimulus were mixed by a professional sound engineer (D.B.), resulting in 68 non-manipulated and 272 manipulated stereo stimuli.

### (c) Audio manipulation algorithms

Contrary to previous studies, which manipulated the complete music ensemble of their stimuli [6,15], we took advantage of professional multi-track recordings and only applied our acoustic manipulations to the ‘lead’ track in each stimulus, before mixing it down with the non-manipulated accompaniment. This applied to vocal tracks in the ‘speech’, ‘screams’, ‘singing’ and ‘singing + accompaniment’ conditions, and to violin tracks in the ‘violin + accompaniment’ condition.

Vocal and violin tracks manipulated in the ‘smiling’ condition underwent a spectral transformation designed to simulate the effect of stretching lips while talking [21]. The transformation extracts the spectral envelope of each successive time frame of the incoming signal, and uses a technique called ‘frequency warping’ to stretch the maxima and minima of this envelope in the 100–5000 Hz frequency band, which loosely correspond to the first five formants of a vocal signal [45]. It then reconstructs the original signal using a phase-vocoder algorithm. In previous work, the transformation was validated to be both natural and effective in simulating the impression of a smiling voice [21,26]. Importantly, like the other two transformations, the procedure can be applied to non-vocal sounds without modification, which allows us to compare the effect of the transformation on vocal (conditions ‘speech’, ‘screams’, ‘singing’, ‘singing + accompaniment’) and non-vocal (condition ‘violin + accompaniment’) tracks. The intensity of the transformation is controlled by multiplicative parameter *α*, used to stretch or compress the signal’s spectral envelope. We applied the smiling transformation in two levels: ‘smile’ (*α* = 1.25), which increased the amount of smile compared with the original, non-manipulated stimuli; and ‘unsmile’ (*α* = 0.85), which decreased the amount of smile.

Vocal and violin tracks manipulated in the ‘vocal tremor’ condition underwent a cyclical pitch-shifting transformation designed to simulate vibrato in afraid/anxious voices (DAVID [22], available open-source at https://forum.ircam.fr/projects/detail/david/). Pitch-shifting denotes the multiplication of the fundamental frequency (*f*_0_) of the original voice signal by a factor *β* (e.g. +25 cents, a 1.5% change of *f*_0_). Here, we apply a periodic modulation of voice *f*_0_, implemented as a sinusoidal modulation of the pitch shift effect with a fixed depth and rate and a small random variation of the rate to increase naturalness. For vocal tremor stimuli in this work, we used a depth of 25 cents, rate of 8 Hz and a randomness parameter of 20%. These parameters were validated in previous work to be both natural and effective in simulating the impression of an anxious voice [22]. Like the other two transformations, the procedure can be applied to either vocal or non-vocal sounds without modification.

Finally, vocal and violin tracks manipulated in the ‘vocal roughness’ condition underwent an amplitude modulation procedure designed to simulate nonlinear phenomena in vocal fold vibration (namely, subharmonics) due to high vocal effort and arousal (ANGUS [23], available open-source at https://forum.ircam.fr/projects/detail/angus). The transformation operates by multiplying the original signal by a lower-frequency modulating signal synchronized on its fundamental frequency (*f*_0_/2), which creates subharmonics at *f*_0_+*f*_0_/2 and *f*_0_−*f*_0_/2, high-pass filtering the resulting subharmonics and mixing them together with the original signal with mixing factor *α* = 1. These parameters were validated in previous work to be both natural and effective in simulating the impression of a negatively aroused voice [23] and, like all others, the procedure can be applied to either vocal or non-vocal sounds without modification. All audio stimuli are available as electronic supplementary material, as well as on https://archive.org/details/smiling_violins.

### (d) Procedure

Participants were presented with pairs of stimuli composed of matched manipulated and non-manipulated versions of the same recording. There were four transformation conditions (68 smile versus non-manipulated pairs; 68 unsmile versus non-manipulated pairs; 68 tremor versus non-manipulated pairs; 68 rough versus non-manipulated pairs) as well as 68 non-manipulated versus non-manipulated control pairs. Presentation order within a pair (manipulated versus non-manipulated, or non-manipulated versus manipulated) was randomized within-participant.

For each pair, participants were asked to evaluate the emotion that was expressed by one recording compared with the other, using a 7-point Likert scale for valence (1 = more negative, 4 = no difference, 7 = more positive) and arousal (1 = more calm, 4 = no change, 7 = more energetic). The order of the comparison within a pair (rating the first recording against the second, or rating the second recording against the first) was fixed within-participant, but counterbalanced between participants. This procedure was the same as in [15].

It is to be noted that results obtained with such an explicit pairwise comparison procedure may differ from those obtained, for example, with single-item rating scales [67] or implicit methods such as the Implicit Association Test [68]. By emphasizing the acoustic difference within pairs, the pairwise method allows us to answer a low-level decoding question (if forced to focus attention on a given acoustic change, what emotional interpretation would that change result in?). Having maximum experimental control over the participant’s locus of attention is important because there are well-known individual- and group-level differences in how people attend to elements in music [69]. Conversely, the pairwise method does not allow us to address questions such as ‘would attention be spontaneously drawn to that feature in a single (unpaired) presentation, compared with other features of the sound?’. Like rating scales, it is also plagued with demand effects, and cannot establish whether such interpretations would be more spontaneously scored as valence/arousal or other untested and potentially non-emotional constructs. We mitigate these effects here by randomizing trials over all manipulations (i.e. having pairs that differ unpredictably on several possible dimensions) and adding control pairs (i.e. pairs with no stimulus difference).

The experiment was divided into three blocks, preceded with a short training block. In the first block participants judged the three musical conditions: ‘singing’, ‘singing + accompaniment’, ‘violin+ accompaniment’. In this block, all stimulus pairs were randomized across conditions. Participants then rated ‘speech’ stimuli in the second block and ‘scream’ stimuli in the third block. The order of these three blocks was fixed for all participants. This procedure (non-music vocal sounds last) was adopted to avoid demand effects where a response strategy learned on speech/screams could then transfer artificially to music stimuli. The procedure leaves the converse risk that participants have learned a strategy on music, and then transferred it to speech and screams, but we alleviated the impact of that possibility on our subsequent interpretations of results by having clear, preregistered hypotheses about the impact of the three manipulations on the latter non-musical stimuli, and finding that these predictions were met.

## Data accessibility

Examples of stimuli, experimental data and analysis code are provided as electronic supplementary material. All stimuli are available at https://archive.org/details/smiling_violins.
